# Why Older Adults Resist Mobile Health Information Services: A Conceptual Model Based on the Technology–Personal–Environment Framework

**DOI:** 10.3390/healthcare14131892

**Published:** 2026-06-29

**Authors:** Ying Zhao, Ziwei Wang, Fan Ke, Xiumei Ma

**Affiliations:** School of Public Administration, Sichuan University, Chengdu 610065, China; zhaoying@scu.edu.cn (Y.Z.); wangziwei@stu.scu.edu.cn (Z.W.); kefanscu@163.com (F.K.)

**Keywords:** mobile health information service, older adults, resistance to use, the TPE framework

## Abstract

**Background/Objectives:** As a key health information and communication technology, mobile health information services (MHISs) play a critical role in delivering health information, enabling remote monitoring, and supporting patient well-being. However, widespread resistance among older adults hinders their access to these information services and undermines these benefits. Employing the technology–personal–environment (TPE) framework, this study constructed and verified a comprehensive model to explain older adults’ resistance to MHIS use. **Methods:** Quantitative data from 430 elderly individuals aged 65 and above from China who participated in the free health check-up basic public health program were analyzed using structural equation modeling. **Results:** Technology access barriers, technology usage barriers, declining physiological conditions, and resistance to change were positively related to technology anxiety. Declining physiological conditions, resistance to change, social legitimacy power, and perceived institutional effort were negatively related to perceived autonomy. Additionally, technology anxiety was positively related to resistance to MHIS use, while perceived autonomy was negatively related to resistance to MHIS use. **Conclusions:** The findings clarify the mechanisms linking technological barriers, individual characteristics, and environmental factors to older adults’ resistance to MHIS use. Therefore, relevant health information service providers should adopt systematic actions that simultaneously alleviate technology anxiety through user-centric design and supportive training while fostering perceived autonomy by respecting older adults’ choices and enabling meaningful participation. These findings offer actionable insights for healthcare information system designers and providers to reduce older adults’ exclusion from digital health information ecosystems, thereby enhancing patient well-being among aging populations.

## 1. Introduction

With the aging population and the rising prevalence of chronic diseases, healthcare demands are increasing rapidly [1,2]. In Europe, for example, healthcare expenditures in several countries exceed 10% of GDP, with some surpassing 15% [3]. The emergence of mobile health information services (MHISs) offers promising solutions to these challenges [4]. By utilizing mobile information communication technologies and devices such as smartphones and tablets, MHIS transcend the spatial and temporal limitations of traditional healthcare delivery [5,6]. Alongside established health information technologies such as Electronic Health Records (EHRs) and Health Information Exchange (HIE) systems, MHIS has emerged as a patient-facing health information communication technology that extends health information access beyond clinical settings, enabling real-time health monitoring, patient education, and remote consultation. Consequently, elderly users’ resistance to MHIS represents not merely a technology adoption failure but a critical barrier to health information access and patient well-being.

Despite the numerous potential benefits of MHIS, significant challenges remain in their practical implementation, particularly among older adults who are generally slower to adopt technological innovations. In developing countries, the adaptation and integration of this emerging healthcare model pose considerable difficulties, which may be associated with the relatively low adoption of MHIS among older adults [7]. This limited uptake not only hinders the development of the mobile health industry but also fails to mitigate the growing healthcare burden associated with population aging. Accordingly, it is essential to investigate the factors that contribute to older adults’ resistance to adopting MHIS.

Given the convenience and accessibility offered by mobile health services, numerous studies have investigated their use among older adults. Existing research has examined factors such as technological facilitators (e.g., perceived usefulness, ease of use, and convenience) [8,9], personal attributes (e.g., self-efficacy and age) [10,11], and external influences (e.g., social support) [12,13], thereby contributing to our understanding of MHIS adoption among older adults. However, several gaps remain in the literature. First, most studies emphasize acceptance and usage, with limited attention to the complex internal mechanisms underlying resistance behaviors. It is important to recognize that adoption and resistance are not simply binary opposites; that is, non-adoption does not necessarily indicate resistance [14]. Second, prior research often adopts a narrow lens, typically focusing on a limited number of dimensions, and lacks an integrated framework that considers technological, individual, and environmental factors simultaneously. Third, while external influences are commonly examined at the community and family levels, the role of healthcare institutions has received insufficient scholarly attention. Additionally, within the context of East Asian or Confucian cultural settings, older adults often hold legitimate social authority. Yet, how this socially conferred power affects their engagement with mobile health information services remains poorly understood.

The Technological–Personal–Environmental (TPE) model provides a comprehensive framework for examining how technological, individual, and environmental factors collectively influence technology acceptance and resistance [15]. Accordingly, this study adopts the TPE framework to investigate how technological barriers (i.e., access and usage), personal factors (i.e., declining physical condition and resistance to change), and environmental elements (i.e., social legitimacy power and perceived institutional effort) affect older adults’ affective and cognitive attitudes—specifically, technology anxiety and perceived autonomy—and ultimately shape their resistance to MHIS use.

This approach yields several important contributions. First, it introduces an innovative application of the TPE framework to examine elderly resistance to MHIS from an integrated technological, personal, and environmental perspective, offering a more holistic understanding of the phenomenon. Second, this study highlights the critical role of technology anxiety and perceived autonomy, revealing their mediating effects in explaining the influence of TPE factors on elderly’s resistance behavior. Third, the study reveals the impacts of social legitimacy and institutional effort, addressing a notable gap in the existing literature regarding social power and organizational influence on resistance behaviors. These findings provide valuable insights for MHIS providers in designing and promoting services that better align with older adults’ needs and preferences.

## 2. Literature Review

### 2.1. Mobile Health Information Services

Mobile health information services (MHISs) refer to the delivery of healthcare services and information through mobile communication technologies, including smartphones, 3G/4G networks, and satellite communications [16]. As a type of health information communication technology, MHIS shares key characteristics with other health information systems such as EHRs and patient portals: they all aim to improve health information flow, enhance patient engagement, and support clinical decision-making. However, MHIS is distinctive in its direct interface with patients, making user resistance uniquely consequential for health information accessibility and patient well-being outcomes [17].

Although MHISs offer numerous advantages and hold significant potential, their successful implementation ultimately depends on the acceptance of the technology and service model by the target population. Existing research consistently indicates that older adults exhibit relatively low acceptance rates of MHISs. To explore low acceptance rates of MHISs, scholars have examined factors across multiple dimensions, including technological factors (e.g., perceived ease of use, perceived usefulness) [18,19,20], personal characteristics (e.g., self-actualization needs, healthcare needs, and health knowledge) [20,21,22], and environmental factors (e.g., social support, subjective norms, and facilitating conditions) [23,24,25,26].

While these studies offer valuable insights into the factors influencing elderly users’ adoption of MHISs, the literature remains limited in direct investigation into resistance to use. It is critical to recognize that adoption and resistance are not merely opposite ends of the same spectrum; non-adoption does not necessarily equate to active resistance [14]. Relying solely on adoption-focused frameworks and variables is insufficient to capture the complex nature underlying resistance behaviors. Moreover, current studies often focused on only one or two dimensions, lacking a comprehensive view on examining elderly’s resistance behavior. This underscores the urgent need for more in-depth research systematically addressing resistance to MHIS use among older adults.

### 2.2. Technology–Person–Environment Model

The Technology–Person–Environment (TPE) model has emerged as a significant theoretical framework in studies of individual technology use [27,28]. It systematically integrates technological factors, personal characteristics, environmental influences, emotions, and attitudes to explain individuals’ intentions and behaviors related to technology adoption [29]. Accordingly, this study adopts the TPE model to examine the underlying mechanisms shaping older adults’ resistance to MHIS use.

#### 2.2.1. Technology Access Barriers and Technology Usage Barriers

In the diffusion of innovative technologies, technical barriers often emerge as critical bottlenecks limiting user use behavior. These barriers can be broadly categorized into two types: technology access barriers and technology usage barriers. Technology access barriers, such as economic costs, device availability, and internet connectivity, directly influence users’ ability to access and engage with the technology [30]. Technology usage barriers, such as interface complexity, cognitive overload, and insufficient technical support, pose challenges during the actual usage process [31]. Among older adults, physiological, psychological, and social factors further exacerbate these challenges, making technological barriers particularly pronounced [32,33]. Accordingly, this study focuses on these two technological barriers.

#### 2.2.2. Declining Physiological Conditions and Resistance to Change

Tummermann emphasized that individuals’ physiological and psychological states significantly affect their willingness to accept new experiences [34]—a relationship that is particularly evident among older adults [35]. Physiologically, aging is associated with a natural decline in functions such as muscle strength, bone density, and cognitive capacity. These changes not only impact daily functioning but also impair the ability to learn and adapt to new technologies [36,37]. Psychologically, as cognitive resources diminish, older adults are more likely to remain within familiar routines. When confronted with unfamiliar technologies, they often exhibit caution or resistance, driven by fear of the unknown or diminished confidence in their ability to use new tools [38,39]. Therefore, this study focuses on two core aspects of the personal dimension: declining physiological conditions and resistance to change.

#### 2.2.3. Perceived Institutional Effort and Social Legitimacy Power

The environment dimension refers to variables related to the broader social context and external conditions influencing individual behavior [40]. Substantial evidence has shown that environmental factors play a critical role in the use of new technologies [41]. Healthcare institutions, as key service providers, offer user training, technical support, and enhanced service experiences. These institutional efforts are essential for shaping older adults’ acceptance and use of mobile health services [42]. However, the role of institutional effort has received relatively limited attention in existing research. Another crucial but underexplored factor is social legitimacy power, which refers to the societal recognition of an individual’s status and the mutual expectations formed through social relationships, norms of fairness, and interdependence [43]. As a senior and digitally marginalized group, older adults often rely on the digital support and normative expectations granted by society to integrate into the digital world and engage with emerging technologies [44]. Therefore, this study focuses on two key environmental variables: perceived institutional effort and social legitimacy power.

#### 2.2.4. Technology Anxiety and Perceived Autonomy

Cognitive and emotional attitudes are frequently regarded as mediating variables within the TPE model [45]. Technology anxiety, as a prevalent negative emotional response, has been widely recognized as a significant inhibitor of technology acceptance [46]. Rajak and Shaw noted that such anxiety often arises from unfamiliarity with new technological features, perceived operational complexity, and doubts about one’s own adaptability [47]. Given the unique physiological, psychological, and social characteristics of older adults, they are especially susceptible to technology anxiety [48]. In parallel, self-determination theory highlights the central role of autonomy in shaping individual motivation and behavior. When individuals perceive a high degree of autonomy, they are more likely to develop positive attitudes and engage proactively with new technologies. Conversely, a perceived lack of autonomy may result in resistance or disengagement [45]. Based on these considerations, technology anxiety and perceived autonomy are considered as the key emotional and cognitive attitudes to MHIS use.

## 3. Research Model and Hypothesis Development

### 3.1. The Roles of Technology Access Barriers and Technology Usage Barriers

Technology access barriers refer to obstacles that limit individuals’ ability to obtain the resources and conditions necessary to access digital technologies and related services. For older adults, such barriers may arise from geographical location, economic constraints, inadequate community network infrastructure, and unfamiliarity with emerging technologies [49]. Technology usage barriers, by contrast, refer to difficulties encountered in operating and using digital technologies after access has been obtained. Even when access is available, older adults may struggle to use online resources and tools effectively because of limited digital skills and lower educational attainment, thereby constraining their ability to fully benefit from digital technologies [50]. According to Cooper [51], these challenges are fundamentally linked to technology anxiety, a psychological state triggered by the perceived gap in technical proficiency between users and others. Jung et al. further found that older adults are particularly susceptible to technology anxiety, as they frequently encounter both access and usage barriers when engaging with digital technology [52]. Given that MHISs are a form of digital technology, such barriers may similarly induce or exacerbate technology anxiety among older adults. Accordingly, this study proposes the following hypotheses:

**H1.**  *Technology access barriers are positively related to technology anxiety.*

**H2.**  *Technology usage barriers are positively related to technology anxiety.*

### 3.2. The Roles of Declining Physiological Conditions and Resistance to Change

Declining physiological conditions involve the deterioration of various bodily functions such as hearing, speech, vision, motor skills, and memory. Meuter et al. found in their study on technology adoption behaviors that compared to younger individuals, older adults are more prone to anxiety due to their declining physiological conditions, which reduces their cognitive adaptability and, consequently, their willingness to use technology [53]. Xue et al. also discovered that the diminished physical condition that comes with aging can lead to a cognitive sluggishness towards emerging technologies, becoming a major concern for older adults when using digital health technologies, thereby provoking anxiety and hesitancy in the face of these technologies [54]. Therefore, the following hypothesis is proposed:

**H3.**  *Declining physiological conditions are positively related to technology anxiety.*

Moreover, declining physiological conditions inevitably result in a reduced capacity for self-care, thereby increasing older adults’ reliance on external assistance [55]. In the context of an increasingly digital society, this dependence is further compounded by the widening digital divide, which exacerbates feelings of disempowerment and loss of control over daily decision-making among older adults [56]. Older adults will frequently experience difficulty in making autonomous decisions that align with their personal preferences and needs, which contributes to diminished perceived autonomy [57]. Therefore, the following hypothesis is proposed:

**H4.**  *Declining physiological conditions are negatively related to perceived autonomy.*

Resistance to change refers to the aversion to and tendency to resist new environments, rules, or demands [58]. According to continuity theory, older adults tend to maintain their past activities and behavioral patterns, showing negative attitudes toward changes [59]. Especially in the case of digital technology application, the discomfort caused by uncertainty can lead to feelings of anxiety, confusion, and panic among older adults [60]. Guo et al. also found that resistance to change can negatively affect older adults’ evaluation of MHIS and trigger technology anxiety [18]. Based on this, the study proposes the following hypothesis:

**H5.**  *Resistance to change is positively related to technology anxiety.*

At the same time, resistance to change is inherently a conservative tendency to maintain the status quo, which can lead to a decrease in an individual’s adaptability when facing new environments or situations [61]. Battistelli et al. revealed a close relationship between resistance to change and perceived autonomy; specifically, if an individual passively adapts to a new environment, this process may enhance their autonomy, whereas strong resistance to change may harm their autonomy [62]. Based on this, the study proposes the following hypothesis:

**H6.**  *Resistance to change is negatively related to perceived autonomy.*

### 3.3. The Roles of Perceived Institutional Effort and Social Legitimacy Power

Perceived institutional effort refers to the user’s subjective perception of the level of effort invested by the provider of a product or service [63]. Users’ perceived effort significantly shapes their overall evaluation and attitudes to use a product or service [64]. For older adults, institutional effort can indeed assist them more efficiently in completing a series of processes on a MHIS. However, it may also overlook older adults’ own initiative, and excessive assistance may lead to over-reliance on institutions. Thus, institutional effort may weaken elderly’s ability to make autonomous choices and behavioral decisions, reducing their perceived autonomy [65]. Based on this, the following hypothesis is proposed:

**H7.**  *Perceived institutional effort is negatively related to perceived autonomy.*

Social power theory suggests that subjects with specific social power can lead objects to comply psychologically or behaviorally [66]. In the Confucian cultural context, older adults are granted a special status due to their age, which is increasingly being recognized as a right within the framework of social support discourse. This social legitimate power is based on the social standing conferred by age and older adults can leverage this legitimate power to seek assistance from society to better adapt to the digital society. When older adults perceive an imbalance between their technical capabilities and system complexity, they strategically emphasize their ‘technological vulnerability’ identity to demand adaptive support from healthcare providers. While this exercise of social power can enhance digital integration, it simultaneously risks fostering excessive dependency on institutional aid, potentially undermining perceived autonomy. Therefore, the following hypothesis is proposed:

**H8.**  *Social legitimacy power is negatively related to perceived autonomy.*

### 3.4. The Role of Technology Anxiety

Technology anxiety refers to the discomfort and irrational anxiety that individuals experience due to the pressure of adapting to digital technology, often manifesting as symptoms such as anxiety, resistance, or panic [67]. In studies of technology adoption and use, technology anxiety is regarded as an important affective variable that reflects users’ psychological responses during human–technology interaction [68]. It has also been used in TAM, UTAUT, and related models to explain adoption barriers, resistance, discontinuance, and avoidance of information technologies [69,70]. As a negative affective response, technology anxiety is closely associated with users’ reluctance to engage with digital technologies [71]. When individuals feel anxious about using digital systems, they may perceive the use process as stressful, uncertain, or difficult to control, which can increase avoidance and resistance [72]. This association is particularly relevant among older adults, whose limited familiarity with digital technologies may make MHIS use more demanding. Prior research has similarly shown that technology anxiety can reduce older adults’ willingness to use health technologies [73]. Based on these findings, we propose the following:

**H9.**  *Technology anxiety is positively related to resistance to MHIS use.*

### 3.5. The Role of Perceived Autonomy

Self-determination theory regards autonomy as one of the basic psychological needs of human beings that shapes individual motivation and behavioral engagement [74]. Collopy further argued that perceived autonomy plays a central role in modern life [75]. As an important psychological resource, autonomy reflects individuals’ sense of control, self-direction, and recognition of their own will in decision-making. For older adults, perceived autonomy remains important despite age-related declines in physical and physiological functions. Retaining autonomy means that they can manage internal emotions, external information, and daily matters independently and proactively without excessive reliance on others [76]. Research has found that elderly individuals with a higher level of perceived autonomy are more willing to actively participate in self-health management and health promotion activities [77]. This positive participation attitude suggests that elderly individuals with strong perceived autonomy may be more likely to actively try to MHIS use. Therefore, the following hypothesis is proposed:

**H10.**  *Perceived autonomy is negatively related to resistance to MHIS use.*

**Figure 1:**
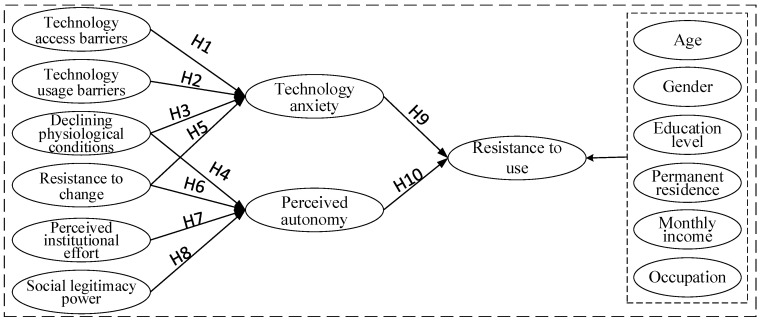
Research model.

In conclusion, this study proposes a research model based on the TPE theoretical framework, as illustrated in Figure 1. To eliminate the influence of demographical variables, we include gender, age, education level, long-term place of residence, personal monthly income, and occupation as control variables.

## 4. Methodology

### 4.1. Research Design

We selected the annual free physical examination program for older adults aged 65 years and above under China’s National Basic Public Health Service Program as the empirical setting of this study. This choice was based on two considerations. First, this program is delivered by primary healthcare institutions and targets older adults aged 65 years and above as part of a nationwide public health service. Therefore, its target population highly overlaps with the general population of older adults in China, indicating that the sample is relevant to and broadly representative of older adults in China. Second, with the digitalization of primary healthcare in China, primary healthcare institutions have introduced mobile health applications to provide older adults with convenient services, including physical examination invitations, appointment scheduling, health condition tracking, and health guidance. However, older adults’ acceptance of these mobile health services remains limited in practice, making this program an appropriate empirical context for examining older adults’ resistance to MHIS use in China.

The research questionnaire is divided into three parts: questionnaire description, basic survey, and measurement of research variables. A 5-point Likert scale is used (1 indicates disagreement and 5 indicates agreement). To ensure the scientific validity of the study questionnaire, we conducted a pre-test questionnaire before the formal distribution. We distributed the designed questionnaire to elderly individuals who have experienced MHISs and interviewed them to identify any statements in the original questionnaire that were difficult to understand or ambiguous. A total of 105 valid questionnaires were collected. The quality of the pre-test survey was confirmed as the Cronbach alpha coefficient for each factor was greater than 0.7, indicating high reliability and validity of the constructs. Based on the feedback from the pre-test participants, we further refined and optimized the questionnaire to ensure its scientific validity for the main survey, and the final measurement scale is presented in Table 1. The model was specified and estimated using IBM SPSS AMOS (Version 28; IBM Corp., Armonk, NY, USA).

### 4.2. Data Collection

The survey targeted individuals aged 65 and older who had participated in the free physical examination basic public health program for older adults and had been in contact with the MHIS. Questionnaires were distributed by visiting various urban and rural senior activity centers, community health service centers, and township health centers across China. Considering the special nature of the respondent group, we specially invited three researchers with no conflict of interest with this study to provide on-site assistance and guidance for older adults in understanding and filling out the questionnaires. A total of 457 questionnaires were collected. After excluding invalid questionnaires due to age ineligibility, incorrect answers, missing answers, and extreme responses, a total of 430 valid questionnaires were retained (all participants volunteered to participate in the survey). This sample size meets Gorsuch’s standard, which suggests that the questionnaire sample size should be at least 5 times the number of items measured, with 10 times or more being even better, allowing for subsequent analysis [88].

## 5. Result Analysis

### 5.1. Demographic Characteristics

The demographic characteristics of the 430 respondents are shown in Table 2. The respondents were distributed across age groups from 65–69 to 90 years or above, with the largest shares in the 65–69 and 70–74 age groups, accounting for 31.9% and 26.7%, respectively. The gender composition was relatively balanced, with males representing 47.9% of the sample and females 52.1%. Educational attainment was concentrated at high school or below, comprising 90.2% of the respondents. Rural residents accounted for 58.1% of the sample, compared with 41.9% from urban areas. Monthly income covered nine categories, with the largest share reporting a monthly income below CNY 500. In terms of occupation, peasants constituted the largest occupational group, accounting for 57.4% of the sample.

### 5.2. Reliability and Validity Analysis

Confirmatory factor analysis (CFA) was conducted to determine whether the indicator variable can be used effectively as a measure of the underlying variable. Convergent validity was primarily assessed using standardized factor loadings, composite reliability (CR), and the extracted average variance (AVE). As shown in Table 3, the standardized factor loadings for all measurement items of the latent variables and the composite reliability (CR) of the latent variables were greater than 0.7, while the AVE was greater than 0.5, indicating that the model has good convergent validity [89].

To assess the discriminative validity, we compared the square root of AVE for a given construct and the correlation coefficients between this construct and other constructs. As shown in Table 4, for each construct, the square root of AVE was greater than the correlation coefficients, suggesting the discriminant validity is acceptable [89].

### 5.3. Multicollinearity and Common Method Bias Tests

Multicollinearity means that the model estimation is distorted or difficult to be accurately estimated due to the existence of exact correlation or high correlation between explanatory variables in the model [90]. Before the validation analysis of structural equation model, a collinearity test of observed variables is required. In this study, the analytical variance inflation factor (VIF) is used to test whether there is multicollinearity between variables. As shown in Table 5, the variance inflation factor (VIF) among the latent variables in the model is all less than 3, which were all lower than the standard of 5.0, indicating that the multicollinearity problem between variables was in a controllable range. Furthermore, as self-reported measures were utilized in the study, Harman’s single-factor test (unrotated exploratory factor analysis) was employed to check for the issue of common method bias (CMB). As the computed one-factor solution value was estimated to be 38.6%, below the suggested threshold of 40%, CMB was not seen as a problem.

### 5.4. Model Fit Indices

In addition, the model fit was assessed using several commonly used fit indices. As shown in Table 6, the results indicated an acceptable model fit, with χ^2^/df = 1.302 and RMSEA = 0.027, both meeting the recommended criteria. Moreover, GFI = 0.919, AGFI = 0.904, CFI = 0.936, NFI = 0.929, IFI = 0.984, and TLI = 0.984, all exceeding the recommended threshold of 0.90 [91]. These results further support that the model demonstrated good fit.

### 5.5. Hypotheses Test

**Figure 2:**
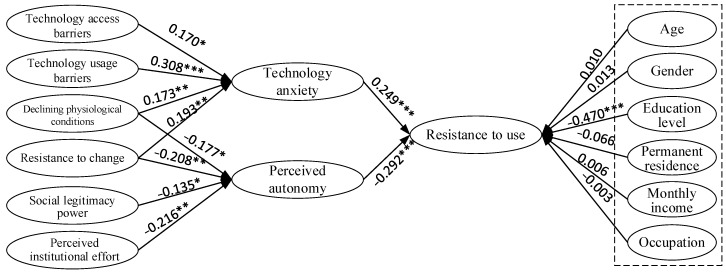
Model results. Note: *** *p <* 0.001, ** *p <* 0.01, * *p <* 0.05.

The path analysis results were shown in Figure 2. Technology access barriers (β = 0.170, *p* < 0.05), technology usage barriers (β = 0.308, *p* < 0.001), declining physiological conditions (β = 0.173, *p* < 0.01), and resistance to change (β = 0.193, *p* < 0.01) were all positively related to technology anxiety. Declining physiological conditions (β = −0.177, *p* < 0.05), resistance to change (β = −0.208, *p* < 0.01), social legitimacy power (β = −0.135, *p* < 0.05), and perceived institutional effort (β = −0.216, *p* < 0.01) were all negatively related to perceived autonomy. Additionally, technology anxiety (β = 0.249, *p* < 0.001) was positively related to resistance to use behavior, while perceived autonomy (β = −0.292, *p* < 0.001) had a significant negative effect on resistance to MHIS use. Furthermore, the results indicate that education level was negatively related to resistance to MHIS use (β = −0.470, *p* < 0.001), while gender, age, residence, monthly income, and occupation were not significantly related to resistance to MHIS use.

### 5.6. Mediating Effects

This study employs the Bootstrapping algorithm to test the mediation effects of technology anxiety and perceived autonomy [92], obtaining confidence intervals at the 95% significance level. The results in Table 7 indicate that both technology anxiety and perceived autonomy play significant mediating roles in the relationships between personal factors, environmental factors and older adults’ resistance to MHIS use. Regarding the technological factors, technology anxiety shows a weak mediating effect on the relationship between technology access barriers and resistance to MHIS use, and no mediating effect between technology usage barriers and resistance to MHIS use.

## 6. Discussion

### 6.1. Key Findings

This study has several key findings. First, the demographic results reveal a cohort-specific background among the respondents. Most were aged between 65 and 84 years, a distribution broadly consistent with the current age structure of the older population in China, while a large proportion had a high school education or below. This educational profile may reflect the limited educational resources and opportunities available to this cohort during their youth, especially before the large-scale expansion of higher education in China. In addition, education level was negatively related to older adults’ resistance to MHIS use, suggesting that older adults with higher educational attainment were less likely to resist mobile health information services. One possible explanation is that higher educational attainment may improve older adults’ ability to understand health information, follow digital service procedures, and adapt to unfamiliar interfaces, thereby reducing resistance when using MHISs. Therefore, strengthening later-life education and digital health literacy support may be important for reducing older adults’ resistance to MHIS use.

Second, technology access barriers and technology usage barriers were positively related to technology anxiety, thereby supporting H1 and H2. These findings align with previous research, which has confirmed that the difficulty of technology acquisition and operational complexity are significant factors contributing to the technology anxiety among older adults [93]. When older adults encounter and attempt to use MHISs, barriers such as lack of internet access, insufficient electronic devices, or issues like unstable network connections, unfriendly user interfaces, and complex functions can directly increase their anxiety towards technology.

Third, declining physiological conditions were positively related to technology anxiety, thereby supporting H3. This finding corroborates previous studies showing that age-related physical decline can intensify older adults’ anxiety toward information technology [30]. Declining physiological conditions were also negatively related to perceived autonomy, thereby supporting H4. This result is consistent with the findings of Perrig-Chiello et al., who suggested that declines in physical abilities may challenge older adults’ independence and confidence in daily life, thereby weakening their perceived autonomy [94]. Additionally, the study also found that resistance to change was positively related to technology anxiety, thereby supporting H5. This result is consistent with previous research indicating that resistance to change is an important factor affecting older adults’ technology anxiety [95]. For older adults who are accustomed to established health service routines, MHISs may be perceived as unfamiliar, disruptive, or difficult to adapt to, thereby increasing technology anxiety. Resistance to change was also negatively related to perceived autonomy, thereby supporting H6. The aversion to and discomfort with new technologies may make older adults feel that their choices and autonomy are limited when faced with emerging entities like MHISs, which aligns with the research of Hwang et al. [96].

Fourth, perceived institutional effort was negatively related to perceived autonomy, thereby supporting H7. This finding is consistent with Kumar et al. [97], suggesting that service providers’ efforts do not always produce the expected positive effects. Although institutions aim to promote mobile health services and help older adults integrate into the digital world, older adults may feel that their agency and autonomy are overlooked during implementation, leading to a sense of marginalization and even triggering resistance. Additionally, social legitimacy power was negatively related to perceived autonomy, thereby supporting H8. In the Confucian cultural context, older adults’ socially recognized age-based status may legitimize their requests for assistance when they encounter difficulties using MHISs. However, repeated activation of this legitimate claim may foster dependence on external support and thereby weaken their perceived autonomy.

Fifth, the technology anxiety was positively related to older adults’ resistance to MHIS use, while perceived autonomy was negatively related to older adults’ resistance to MHIS use, thereby supporting H9 and H10. In line with previous research, technology anxiety, as a negative emotion, may weaken older adults’ willingness to accept new technologies [73]. By contrast, perceived autonomy, as a positive psychological factor, may strengthen older adults’ willingness to engage with MHISs. When older adults have a strong sense of autonomy, confidence in their ability to use digital services, and the perception that they can make independent choices, they may be more inclined to try and accept MHIS. In addition, the mediation results further indicate that technology anxiety and perceived autonomy are important emotional and cognitive mechanisms linking TPE factors to older adults’ resistance to MHIS use.

### 6.2. Theoretical Implications

This study yields several theoretical implications. Firstly, in contrast to most previous studies that focused on the positive aspects of the adoption behavior of mobile medical applications by elderly users, this study discusses the resistance to use of mobile medical services. Utilizing the TPE theoretical framework, we systematically analyzed the impacts of technology, the individual, and the environment factors, providing a comprehensive view on elderly’s resistance to MHIS use. Ultimately, these results deepen our understanding of the resistance to MHIS and expand the application boundaries of the TPE theory in the study of elderly users’ behavior.

Secondly, previous research on negative behaviors has often focused solely on the role of negative emotions [22], but neglected the potential impact of positive attitudes like perceived autonomy. Adopting an integrative perspective, this study identifies the mediating roles of both technology anxiety and perceived autonomy. This not only enhances our understanding of the psychological mechanisms behind the resistance to MHIS use for older adults but also provides a more complex and refined theoretical framework on examining technology acceptance and resistance for the future research.

Finally, this study fills a gap in understanding how social legitimacy power and institutional effort shape the elderly’s behavior towards MHISs. Although the role of social power and healthcare institutions in promoting health services are crucial, this area has been largely overlooked in research. Especially, the study expands empirical research on the potential negative impacts of institutional efforts, echoing on the findings of Kumar, Venkatesan [97] in the field of marketing—that the efforts of service providers do not always yield the expected positive effects. These findings not only provide a new understanding of the unintended negative effects of institutional efforts but also further deepen our reflection on the complex relationship between institutional efforts and ideal outcomes.

### 6.3. Practical Implications

The findings of this study offer some practical insights. First, technology access barriers and technology usage barriers play a pivotal role in older adults’ resistance to using MHISs by increasing uncertainty and technology anxiety during use. To reduce technology access barriers, primary healthcare institutions should provide basic digital access support, such as clear information about MHISs, staff-assisted first-time use, and guidance on obtaining stable access to smart devices and internet services. To address technology usage barriers, the design of mobile health apps should focus on simplicity and ease of use, eliminating unnecessary complex features and frequent system updates to make it easier for older users to get started. In addition, the practicality and daily interactivity of mobile health apps should be enhanced by developing modules suitable for older adults’ daily needs and interactions [93], such as health education, wellness guidance, and social interaction, to create an engaging and useful online community environment. This supportive digital environment may help increase sustained engagement among older users and enhance their health awareness and psychological resilience through continuous health knowledge dissemination and psychological support, thereby alleviating technology anxiety.

Second, declining physiological conditions and resistance to change are two significant factors contributing to older adults’ reluctance to embrace new technology. As people age, they inevitably face these challenges and may experience technology anxiety when adapting to new digital services. Therefore, it is necessary to implement effective measures to support and motivate their adaptation to new technology. For instance, offering personalized training and coaching services tailored to the needs and characteristics of older adults can help them gradually overcome their fear and discomfort with new technology. Additionally, establishing a professional and patient customer service team ready to address various questions and difficulties encountered by elderly users ensures a smooth experience with MHISs.

Third, while the importance of perceived institutional effort cannot be overlooked, research indicates that it does not always yield the expected positive results. Sometimes, excessive intervention or inappropriate support can backfire. Therefore, service institutions need to carefully assess whether their efforts truly align with the needs and preferences of older adults, avoiding over-intervention or unnecessary pressure. It is also essential to listen to and understand the feedback from older adults, continuously optimizing service strategies and methods to ensure that the support provided meets their actual needs while respecting their right to make autonomous choices.

Fourth, service providers should fully respect the autonomy and individual needs of older adults when designing products and services, giving them more choices and control. For example, allowing users to customize interface layouts and set reminder notifications can make older adults feel that they are in charge rather than passively receiving services. Moreover, collecting feedback through user satisfaction surveys and making adjustments based on this information can ensure continuous improvement in service quality.

## 7. Conclusions, Limitations and Future Research

### 7.1. Conclusions

Based on the TPE theoretical framework, this study developed a conceptual model examining factors related to resistance to MHIS use among older adults. The findings indicate the following: (1) technology access barriers, technology usage barriers, declining physiological conditions, and resistance to change are positively related to technology anxiety; (2) declining physiological conditions, resistance to change, social legitimacy power, and perceived institutional effort are negatively related to perceived autonomy; (3) perceived autonomy is negatively related to resistance to MHIS use, whereas technology anxiety is positively related to resistance to MHIS use. This study offers novel empirical insights into factors related to resistance to MHIS use among older adults and extends the application scope of the TPE framework within digital health adoption research.

### 7.2. Limitations and Future Research

Although this research has yielded valuable insights, certain limitations remain. First, the study sample was limited to older adults in China, with a relatively limited sample size. Future research should consider broadening the scope to include elderly populations from diverse international contexts to enhance the generalizability and external validity of the findings. Second, this study examined factors associated with older adults’ resistance to MHIS use but did not further distinguish their specific manifestations. Future research could further refine these factors and incorporate additional individual characteristics, such as digital literacy and prior technology experience, to provide a more nuanced understanding of how these factors influence older adults’ resistance to MHIS use. Third, this study employed a cross-sectional survey design, which limited the causal inference. Future research could adopt longitudinal designs to examine how older adults’ resistance to MHIS use develops and changes over time.

## Figures and Tables

**Table 1:** Design scale.

Construct	Item	Questions	References
Technology Access Barriers	TAB1	It is difficult for me to get information about technology	[78]
	TAB2	I hardly ever use a smartphone or other smart device (e.g., iPad, computer, etc.)	
	TAB3	I think it is difficult to use the Internet whenever I want	
Technology Usage Barriers	TUB1	I find it challenging to browse information on smart devices (like small font, complex interfaces, etc.)	[79,80]
	TUB2	I often do not know how to solve it when I make a mistake while operating them	
	TUB3	I often forget certain steps to operate the smart device	
	TUB4	I do not quite understand the meaning of some symbols or buttons in mobile health apps	
	TUB5	I find it difficult to use smart devices because the interface always changes (like pop-ups, screen transitions, etc.)	
Declining Physiological Conditions	DPC1	My physical condition requires me to put in more effort for daily activities	[81]
	DPC2	My physical condition limits the types of activities I can do	
	DPC3	My physical condition makes my daily activities difficult	
Resistance To Change	RTC1	I do not want mobile health apps to change the way I deal with health-related issues (like switching from in-person appointments to online scheduling, or from receiving notifications via phone or text to checking and getting information through mobile health services)	[18,21]
	RTC2	I do not want mobile health apps to change the way I maintain my health	
	RTC3	I do not want mobile health apps to change the way I interact with others	
	RTC4	Overall, I do not want mobile health apps to change my current lifestyle	
Perceived Institutional Effort	PIE1	In the promotion of Mobile health information services, the staff of medical institutions have paid a lot of energy	[63,82]
	PIE2	In the promotion of Mobile health information services, the staff of medical institutions are persistent	
	PIE3	In the promotion of Mobile health information services, the staff of medical institutions spend a lot of time in the service process	
	PIE4	In the promotion of Mobile health information services, the staff of medical institutions have made great efforts in the service process	
Social Legitimacy Power	SLP1	Based on my standpoint, I can ask others for help with mobile health information services	[83,84]
	SLP2	I have the right to ask others to offer me help with mobile information health services	
	SLP3	As an elderly person, I believe it is reasonable to expect support from others when I need it, so that I am not completely dependent on myself using mhealth apps for information and services	
Technology Anxiety	TA1	Using mobile health information services makes me anxious	[54,85]
	TA2	I get scared when I think about the negative consequences that might result from a mistaken operation on smart devices	
	TA3	I am unwilling to use technology because I fear making mistakes that I cannot correct	
	TA4	Using mobile health information services makes me nervous	
Perceived Autonomy	PA1	I can decide independently when to use mobile information health services	[74,86]
	PA2	I can decide independently where to use mobile information health services	
	PA3	I can decide independently which services to use with mobile health information services	
	PA4	I can decide for myself which services provided by mobile health information services to use	
	PA5	I can autonomously use the mobile information health service to get the services I need	
Resistance To Use	RTU1	I prefer to receive medical help or health information services through other means rather than using mobile information health services	[87]
	RTU2	I do not have any plans to use mobile information health services for now	
	RTU3	I do not want to use mobile health information services to deal with my health issues	

**Table 2:** Demographic characteristics of the respondents.

Variables	Category	Frequency	Percentage (%)
Age	65–69	137	31.9
	70–74	115	26.7
	75–79	80	18.6
	80–84	59	13.7
	85–89	28	6.6
	61 or older	11	2.6
Gender	Male	206	47.9
	Female	224	52.1
Education level	High school or lower	388	90.2
	College	42	9.8
Permanent residence	Urban	180	41.9
	Rural	250	58.1
Monthly income	Less than 500	133	30.9
	501–1000	67	15.6
	1001–1500	41	9.5
	1501–2000	36	8.4
	2001–2500	22	5.1
	2501–3000	19	4.4
	3001–3500	26	6.0
	3501–4000	28	6.5
	above than 4000	58	13.5
Occupation	Scientific, educational, cultural, medical and health professionals	33	7.7
	Leading cadres of state organs, Party and mass organizations, state-owned enterprises and public institutions	21	4.9
	Ordinary public officials in state organs, Party and mass organizations, state-owned enterprises and public institutions	45	10.5
	soldier	5	1.2
	Peasant	247	57.4
	Worker	37	8.6
	Business and service workers	12	2.8
	Freelance work	20	4.7
	other	10	2.3

**Table 3:** Reliability and convergent validity.

Construct	Item	Cronbach’s Alpha	Std	AVE	CR
TAB	TAB1	0.843	0.810	0.912	0.675
	TAB2		0.845		
	TAB3		0.752		
TUB	TUB1	0.926	0.804	0.882	0.713
	TUB2		0.870		
	TUB3		0.858		
	TUB4		0.843		
	TUB5		0.853		
DPC	DPC1	0.860	0.832	0.860	0.672
	DPC2		0.827		
	DPC3		0.800		
RTC	RTC1	0.900	0.826	0.901	0.694
	RTC2		0.812		
	RTC3		0.855		
	RTC4		0.838		
PIE	PIE1	0.901	0.825	0.902	0.696
	PIE2		0.813		
	PIE3		0.835		
	PIE4		0.864		
SLP	SLP1	0.775	0.754	0.774	0.534
	SLP2		0.701		
	SLP3		0.736		
TA	TA1	0.887	0.779	0.887	0.663
	TA2		0.842		
	TA3		0.843		
	TA4		0.790		
PA	PA1	0.911	0.818	0.911	0.673
	PA2		0.831		
	PA3		0.856		
	PA4		0.824		
	PA5		0.769		
RTU	RTU1	0858	0.751	0.858	0.669
	RTU2		0.872		
	RTU3		0.826		

**Table 4:** Discriminant validity.

	TAB	TUB	DPC	RTC	PIE	SLP	TA	PA	RTU
TAB	0.822								
TUB	0.678	0.844							
DPC	0.554	0.567	0.820						
RTC	0.562	0.56	0.642	0.833					
PIE	0.62	0.681	0.606	0.557	0.834				
SLP	0.543	0.527	0.476	0.547	0.553	0.731			
TA	0.585	0.63	0.567	0.573	0.528	0.443	0.814		
PA	−0.423	−0.436	−0.506	−0.516	−0.514	−0.454	−0.394	0.820	
RTU	0.312	0.329	0.336	0.342	0.329	0.284	0.417	−0.463	0.818

**Table 5:** Multicollinearity test.

	RTU	TA	TAB	TUB	DPC	RTC	PIE	PA	SLP
RTU									
TA	1.246								
TAB		1.735							
TUB		1.806							
DPC		1.654						1.685	
RTC		1.700						1.701	
PIE								1.624	
PA	1.245								
SLP								1.399	

**Table 6:** Fit indices for the model.

Indicators	RTU	TA	TAB	TUB	DPC	RTC	PIE	PA
Fitting indicators	1.302	0.027	0.919	0.904	0.936	0.929	0.984	0.984
Reference standard	1–3	<0.05	>0.90	>0.90	>0.90	>0.90	>0.90	>0.90

**Table 7:** Mediating effects test results.

Paths	Total Effects	Direct Effects	95% Confidence Interval		Indirect Effect	95% Confidence Interval	
			Lower	Upper		Lower	Upper
Technology access barriers → technology anxiety → resistance to use	0.348 ***	0.274 ***	0.176	0. 392	0.073 *	0.017	0.142
Technology usage barriers → technology anxiety → resistance to use	0.409 ***	0.357 ***	0.255	0.467	0.051	−0.013	0.124
Declining physiological conditions → technology anxiety → resistance to use	0.295 ***	0.177 ***	0.07	0.307	0.118 ***	0.06	0.191
Resistance to change → technology anxiety → resistance to use	0.280 ***	0.158 **	0.045	0.281	0.122 ***	0.062	0.201
Declining physiological conditions → perceived autonomy → resistance to use	0.292 ***	0.156 **	0.055	0.266	0.136 ***	0.085	0.203
Resistance to change → perceived autonomy → resistance to use	0.279 ***	0.139 **	0.037	0.245	0.140 ***	0.087	0.211
Social legitimacy power → perceived autonomy → resistance to use	0.341 ***	0.200 ***	0.092	0.327	0.141 ***	0.087	0.209
Perceived institutional effort → perceived autonomy → resistance to use	0.359 ***	0.215 ***	0.105	0.330	0.144 ***	0.085	0.218

Note: *** *p <* 0.001, ** *p <* 0.01, * *p <* 0.05.

## Data Availability

The data presented in this study are available on request from the corresponding author due to privacy restrictions.
